# Environmental enrichment strengthens corticocortical interactions and reduces amyloid-β oligomers in aged mice

**DOI:** 10.3389/fnagi.2014.00001

**Published:** 2014-01-23

**Authors:** Marco Mainardi, Angelo Di Garbo, Matteo Caleo, Nicoletta Berardi, Alessandro Sale, Lamberto Maffei

**Affiliations:** ^1^Neuroscience Institute of the National Research CouncilPisa, Italy; ^2^Biophysics Institute of the National Research CouncilPisa, Italy; ^3^Department of Neuroscience, Psychology, Drug Research and Child Health (NEUROFARBA), University of FlorenceFlorence, Italy; ^4^Accademia dei LinceiRoma, Italy

**Keywords:** environmental enrichment, aging, cross-correlation, EEG, vGluT-1, vGAT, neprilysin, amyloid-β oligomers

## Abstract

Brain aging is characterized by global changes which are thought to underlie age-related cognitive decline. These include variations in brain activity and the progressive increase in the concentration of soluble amyloid-β (Aβ) oligomers, directly impairing synaptic function and plasticity even in the absence of any neurodegenerative disorder. Considering the high social impact of the decline in brain performance associated to aging, there is an urgent need to better understand how it can be prevented or contrasted. Lifestyle components, such as social interaction, motor exercise and cognitive activity, are thought to modulate brain physiology and its susceptibility to age-related pathologies. However, the precise functional and molecular factors that respond to environmental stimuli and might mediate their protective action again pathological aging still need to be clearly identified. To address this issue, we exploited environmental enrichment (EE), a reliable model for studying the effect of experience on the brain based on the enhancement of cognitive, social and motor experience, in aged wild-type mice. We analyzed the functional consequences of EE on aged brain physiology by performing *in vivo* local field potential (LFP) recordings with chronic implants. In addition, we also investigated changes induced by EE on molecular markers of neural plasticity and on the levels of soluble Aβ oligomers. We report that EE induced profound changes in the activity of the primary visual and auditory cortices and in their functional interaction. At the molecular level, EE enhanced plasticity by an upward shift of the cortical excitation/inhibition balance. In addition, EE reduced brain Aβ oligomers and increased synthesis of the Aβ-degrading enzyme neprilysin. Our findings strengthen the potential of EE procedures as a non-invasive paradigm for counteracting brain aging processes.

## Introduction

The increase in human lifespan has led to a rise in the prevalence of age-related pathologies, such as Alzheimer's disease (AD) and other forms of dementia (Wimo and Prince, 2010). Reduced functional connectivity (Ystad et al., 2011), leading to reduced corticocortical interactions, in particular between primary sensory areas (Golob et al., 2001; Stephen et al., 2010) takes place during brain aging and can be a major contributor to the functional impairment observed in AD (D'Amelio and Rossini, 2012). Deposition of insoluble aggregates of amyloid-β protein (Aβ) (Blessed et al., 1968) can be a molecular substrate of the functional impairment observed in aging and AD, with more recent studies pointing to a critical role of soluble Aβ oligomers as the prime cause of synaptic dysfunction related to dementia (McLean et al., 1999; Mucke et al., 2000; Shankar et al., 2009). Notably, deposition of Aβ oligomers and plaques takes place in the neocortex, also during non-pathological aging (Bouras et al., 1994), and then evolves into clinical manifestations (Rodrigue et al., 2012).

Another important aspect of brain aging is the variation in intracortical inhibition. Experimental data addressing this point in humans are contrasting. On the one hand, clinical electrophysiology show a decrease in paired-pulse suppression of magnetoencephalography (MEG) responses in the somatosensory cortex (Cheng and Lin, 2013). Similarly, a reduction in short-interval intracortical inhibition (SICI) of electromyographic (EMG) responses to pair of stimuli delivered to the cortex by transcranial magnetic stimulation (TMS) has been reported (Marneweck et al., 2011). On the other hand, findings from similar experiments using TMS and EMG indicate a progressive increase in SICI with age (McGinley et al., 2010). In an analogous manner, experiments performed on rats indicate an age-related decline in intracortical inhibition, measured with electrophysiological recordings on brain slices (Schmidt et al., 2010), whereas a higher frequency of inhibitory postsynaptic currents (IPSCs) was observed in patch-clamp recordings on slices from aged rhesus monkey prefrontal cortex in comparison to young controls (Luebke et al., 2004).

Accumulating evidence from human studies shows that brain health in elderly people can be substantially maintained by adopting a physically and mentally active life style, thus delaying age-related decline and decreasing neurodegeneration (Selkoe, 2012). However, the precise mediators of this protective process are not known in detail. Environmental Enrichment (EE), an experimental protocol consisting in enhanced motor activity, social interaction opportunities and increased cognitive stimulation (Van Praag et al., 2000), has been widely used in laboratory models to investigate the impact of sensorimotor stimulation on brain functioning and the underlying mechanisms. Substantial EE effects on brain physiology and pathology have been demonstrated in a variety of conditions (Nithianantharajah and Hannan, 2006), and have been shown to be strongly related to the induction of neural plasticity processes (Sale et al., 2009).

Here, we exploited EE as a model for enriched lifestyle conditions in order to investigate how it would affect neural activity, corticocortical interactions, expression of plasticity markers and amyloid levels in aged, wild-type mice.

We report that exposure to EE modifies the power spectra of local field potentials in the primary visual and auditory cortices (V1 and A1, respectively), increases their cross-correlation across the whole range of EEG frequency bands, modulates the intracortical inhibition/excitation balance, reduces cortical levels of endogenous Aβ soluble oligomers, and increases the expression of neprilysin, one of the main enzymes regulating amyloid protein catabolism.

## Materials and methods

### Animals and ethics statement

All procedures complied with Italian Ministry of Health (Law 116/92) and European Communities Council (Directive 86/609/EEC) guidelines.

C57BL/6J male mice were housed in an animal room with a 12h/12h light/dark cycle, with food and water available *ad libitum*. The standard rearing condition (SC) consisted of a 26 × 18 × 18 cm cage housing 3 animals. The environmental enrichment condition (EE) consisted of a large cage (44 × 62 × 28 cm) containing one running wheel for voluntary physical exercise and differently shaped objects (tunnels, shelters, stairs) that were repositioned twice a week and completely substituted once a week. Six to eight mice were housed in EE cages. Aged mice (age 17 months, EE-OLD group) were placed in EE for one month after electrode implantation, then brain samples were collected (see below). Age-matched mice reared in SC were used as controls (SC-OLD group). For the experiments on young mice (EE-YOUNG and SC-YOUNG groups), pregnant dams were put either in EE 1 week before delivery together with 2–3 non-pregnant helper females, or left in SC, in the latter case with no helper females. Pups were weaned at postnatal day (P) 25.

### Neuronatomical tracing

Monosynaptic connections between the primary visual (V1) and auditory cortices (A1) were identified using the retrograde tracer Cholera Toxin β subunit (CTB, Sigma, USA). Mice were mounted on a stereotaxic apparatus, then a burr hole was made in the skull overlying V1. Injections were performed in the core of V1, 0.0 mm anteroposterior (AP) and 2.5 mm lateral (L) to the lambda point (Paxinos and Franklin, 2013). Only 50 nl of CTB solution (1% in water) at 600 μm depth were delivered using a 1 μl Hamilton syringe (Hamilton, USA) and a glass pipette, thus avoiding spilling of CTB outside of V1. After allowing 3 days for CTB transport to neuronal somata and processes, animals received terminal anaesthesia with chloral hydrate and transcardial perfusion with 25 ml of PBS followed by 50 ml of 4% PFA in 0.1 M phosphate buffer pH 7.4 (PB). Brains were quickly removed, cryoprotected in 30% W/V sucrose in PB for 3 days, then 50 μm-thick coronal sections were obtained with a sliding microtome (Leica, Germany). CTB labeling was visualized by immunohistochemistry. Free-floating sections were blocked in 5% normal rabbit serum (NRS), 2.5% bovine serum albumin (BSA), 0.3% Triton X-100 in PBS for 2 h at RT. The primary antibody was 1:4000 anti-CTB made in goat (Calbiochem, USA), in 2% NRS, 2.5% BSA, 0.1% Triton X-100 in PBS, overnight at 4°C. The secondary antibody was 1:400 donkey anti-goat conjugated to Alexa-568 (Molecular Probes, USA) in 2% NRS, 2.5% BSA, 0.1% Triton in PBS. Incubation lasted 2.5 h at RT, then nuclei were counterstained with 1:5000 YoYo-1 (Invitrogen, USA) in PBS. Sections were mounted on glass slides and protected with VectaShield (Vector Labs, USA). Images were acquired with a confocal microscope (Leica) using a 10X objective. Five SC and three EE animals were used for neuroanatomical tracings.

### Chronic electrode implants and LFP recordings

Local field potential (LFP) recordings were performed in awake, freely moving mice using an adaptation of the protocol described by Mainardi et al. (2012).

Under avertin anaesthesia (0.01 ml/g) and after placement in a stereotaxic apparatus, the skull was exposed and four burr holes were drilled (see Figure 2 and below), paying attention not to damage the dural surface. Four 120 μm-thick nichrome wire electrodes and an insulated copper ground cable were soldered to a multipin socket. This device was held by an adjustable manipulator and the electrodes were positioned to obtain an electrical contact without lesioning the dura mater. LFPs were sampled by placing in each cortical area two electrodes, spaced by 1.0 mm, to detect local electrical activity between the two sites. A screw was positioned on the occipital bone and connected with the ground cable, while an additional screw was installed on the frontal bone for improved stability. The implant was fixed with acrylic cement (Paladur, Pala, Germany). Stereotaxical coordinates were (i) 2.0 mm and 3.0 mm L and 0.0 mm AP to lambda for V1; (ii) 3.9 mm L and −2.0 and −3.0 mm AP to bregma for primary auditory cortex (A1) (Figure 4) (Paxinos and Franklin, 2013). Five days were allowed for recovery from surgery.

After a 1 h habituation to the test cage, LFPs were recorded for 1 h using a digital acquisition system, composed of a custom-made buffer to eliminate movement artifacts, an amplifier and an acquisition card (National Instruments, USA), plugged via USB to a computer. The custom-made acquisition software was based on LabView (National Instruments). Cortical LFPs were sampled at 100 Hz as the differential between two adjacent electrode sites, 10000× amplified and 0.3–45 Hz band-passed.

### LFP analysis

#### Data description

Data sets consisted of bivariate time series corresponding to LFPs simultaneously recorded from V1 and A1. Frequency band decomposition of LFP signals was performed considering the main EEG frequency bands: δ (0.3–4 Hz), θ (4–8 Hz), α (8–12 Hz), β (12–30 Hz) and low-γ (30–45 Hz), which were isolated by band-pass filtering the original LFP signal using a custom-made application based on LabView. Each time series was normalized to zero mean and unit standard deviation. To satisfy the request of stationarity all-time series were partitioned in half-overlapping windows.

#### Spectral analysis

Spectral analysis of LFPs was performed using the Fast Fourier Transform (FFT) algorithm (Bendat and Persol, 1971). LFP series were divided in half-overlapping windows of 8192 data points and FFT was computed on each segment. Bartlett windowing was used to reduce the effects of leakage on the power spectrum. Then, the total spectral power (PWT) was estimated for each window. The contribution of each frequency band to the total power was evaluated as: PWT(band)/PWT, where PWT(band) is the corresponding spectral power of a given band. For each experimental group, the set of all values of PWT and PWT(band)/PWT were obtained, then mean ± s.e.m. values were computed.

#### Cross correlation

The correlation level of LFP signals from V1 and A1 was quantified using a measure based on the cross-correlation function (CC) (Mormann et al., 2003), with an adaptation of the protocol we described in Di Garbo et al. (2011).

Let (*x*_*i*_, *y*_*i*_), *i* = 1, … *N* be two discrete signals, then CC at time lag *n*Δ*t*_*s*_ is defined as:

$$\rho (n)=\sum_{i=1}^{N-n}\left(x_{i+n}-\bar{x})(y_{i}-\bar{y}\right)/\left(\sqrt{\sum_{i=1}^{N}{\left(x_{i}-\bar{x}\right)}^{2}}\sqrt{\sum_{i=1}^{N}{\left(y_{i}-\bar{y}\right)}^{2}}\right)$$

where Δ*t*_*s*_ is the sampling interval of the signals and *x*, *y* are the mean values of *x*_*i*_ and *y*_*i*_.

To quantify the interdependence properties between the LFPs in V1 and A1, we employed the following measure:

$$\rho_{m}=\underset{j}{\max}\left|\rho (j)\right|$$

where *j* = 1, 2, .., *N*_Lag_ is the number of time lags. ρ_*m*_ quantifies the higher value of the linear correlation between two signals over the time windows *N*_Lag_ Δ*t*_*s*_. The adopted value for *N*_Lag_ was 3, which represents a compromise between computational advantage and the choice of a physiological time window. For each experimental group the set of all values of ρ_*m*_ was obtained; then, the corresponding mean ± s.e.m. values were calculated.

The CC analysis described above was employed both on the original LFP signals (0.3–45 Hz) and on the δ, θ, α, β, and low-γ bands (see above, Data Description).

#### Western blotting

After chloral hydrate anaesthesia, the brain was removed from the skull and the entire cortical mantle was dissected out, frozen in dry ice and stored at −80°C. Samples were homogenated and soluble proteins were extracted with a lysis buffer adapted from Lesne et al. (2006), that contained 0.01% NP-40, 0.1% SDS, 50 mM TrisHCl, pH 7.6, 150 mM NaCl, 2 mM EDTA, 0.1 mM Na_3_VO_4_, 1 μg/ml leupeptin, 1 μg/ml aprotinin, and 1 mM PMSF. The total protein concentration was assessed with a bovine serum albumin-based Bradford assay kit (Bio-Rad, USA). Non-boiled protein extracts were loaded on Tris-HCl 12% or 4–12% precast gels (Bio-Rad) and separated using SDS-PAGE (1 h at 200 V), then blotted on nitrocellulose membrane (Bio-Rad). Blots were blocked with 5% milk and 0.2% Tween-20 in TBS, for 2 h at RT. Primary antibodies were diluted in 2.5% milk, 0.1 Tween-20 (see Table 1 for antibodies and dilutions). Incubation lasted overnight at 4°C. Blots were then incubated with HRP-conjugated goat anti–rabbit or goat anti-mouse secondary antibodies (Jackson Labs, USA), for 2 h at RT. The signal was detected by enhanced chemiluminescence (Immun-Star western C, Bio-Rad) and autoradiography films (HyperFilm, GE Healthcare, USA). As an internal quantification standard, blots were also probed for α-tubulin. To do this, blots were blocked again for 30 min at RT adding 0.1% sodium azide to extinguish the peroxidase activity of the previously incubated secondary antibody. Then, blots were incubated as described above with 1:10000 anti-α-tubulin mouse monoclonal antibody (Sigma, Germany) or 1:15000 anti-α-tubulin rabbit polyclonal antibody (AbCam, UK) for 45 min at RT. Then, blots were reacted with 1:20,000 HRP-conjugated goat anti-mouse or goat anti-rabbit secondary antibodies. Quantification of blots was performed using the ImageStudio software (Li-Cor, USA).

**Table 1:** **Antibodies, dilutions and amount of protein loaded in Western blot assays**.

**Protein probed**	**Protein extract loaded (μg)**	**Primary antibody**	**Dilution**	**Secondary antibody**	**Dilution**
vGluT-1	10	Synaptic Systems 135303	1:4000	HRP-conjugated goat anti-rabbit	1:20000
vGAT	15	Synaptic Systems 131003	1:1000	HRP-conjugated goat anti-rabbit	1:20000
Aβ oligomers	30	Millipore A11 AB9234	1:10000	HRP-conjugated goat anti-rabbit	1:40000
Aβ oligomers	10	Covance 4G8 SIG-39200	1:2000	HRP-conjugated goat anti-mouse	1:30000
Neprilysin	10	AbCam ab951	1:1000	HRP-conjugated goat anti-mouse	1:20000

#### Statistical analyses

For analysis of LFP signals, a total of 35 mice were used, 13 for the EE-OLD group, 10 for the SC-OLD group, 6 for the EE-YOUNG group and 6 for the SC-YOUNG group. For comparing the results of Fourier analysis (Figure 3 and Figure S1), Three-Way ANOVA followed by Holm-Sidak *post-hoc* test was used, with factors “rearing” (with levels EE and SC), “age” (with levels AGED and YOUNG) and “cortical area” (with levels A1 and V1). For comparing the results of Cross correlation analysis (Figure 4 and Figure S2), Two-Way ANOVA followed by Holm-Sidak *post-hoc* test was used.

For Western blots on vGluT-1 and vGAT (Figure 5), a total of 26 mice were used, 12 for the EE-OLD group, 9 for SC-OLD group and 5 for the SC-YOUNG group. Statistical significance was assessed using One-Way ANOVA followed by Tukey *post-hoc* test.

For Western blots on amyoid-β oligomers and neprilysin (Figures 6, 7), a total of 36 mice were used, 22 for the EE-OLD group and 14 for the SC-OLD group. Statistical significance was assessed using Student's *t*-test.

All the analyses have been performed using the SigmaPlot 12 software (SyStat Software, USA).

Data are presented as mean ± s.e.m.

## Results

### Identification of monosynaptic connections between the primary visual and auditory cortex

**Figure 1:**
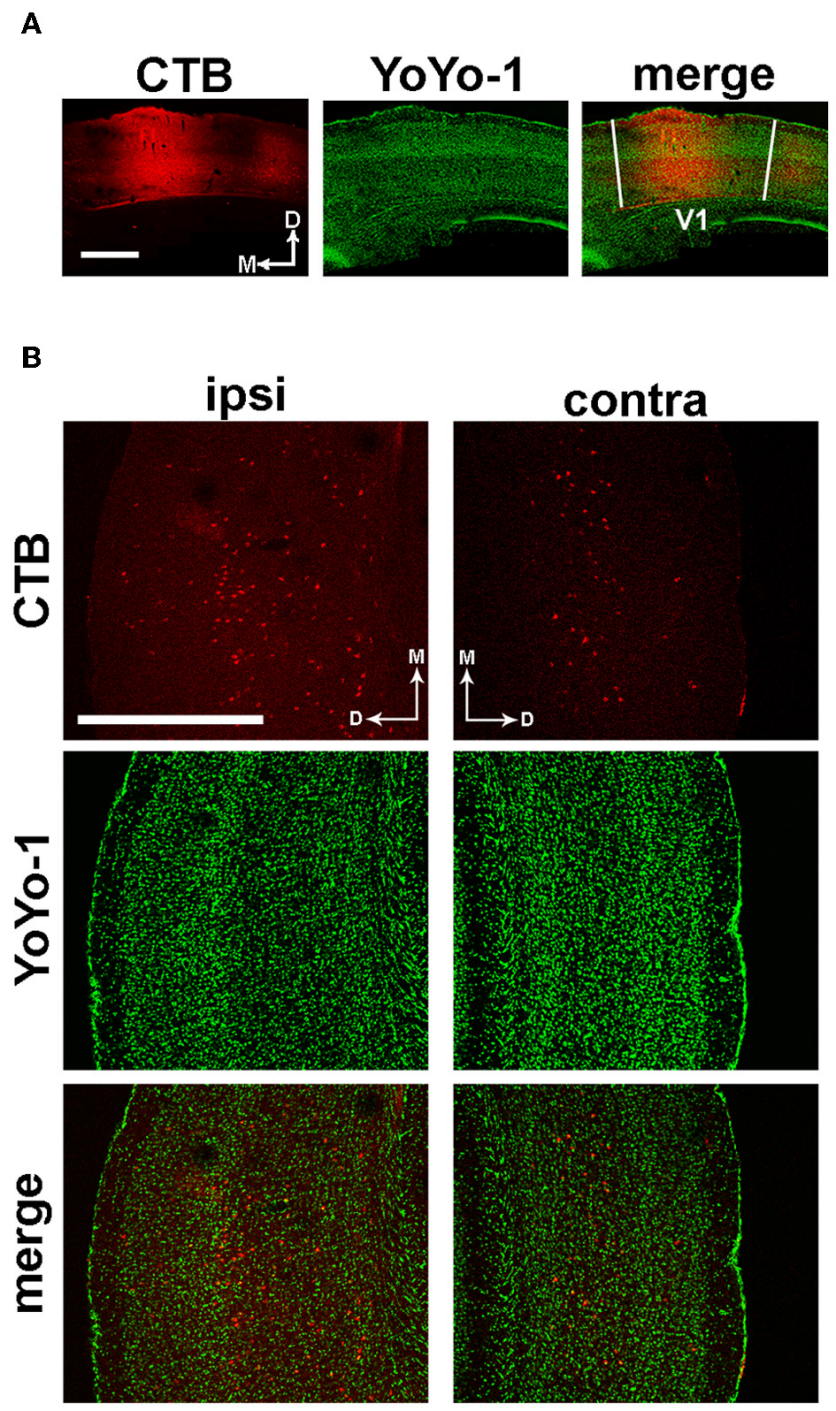
**Identification of anatomical projections from A1 to V1. (A)** Representative image of the CTB injection site and YoYo-1 nuclear counterstaining, which remained confined within the boundaries of V1. **(B)** Left; immunofluorescence images showing retrograde transport of CTB injected in V1 to ipsilateral layers IV-VI of the primary auditory cortex (red), with a few scattered somata in layer II-III. Right; in the hemisphere contralateral to the injection site, a few cells are labeled in layers V–VI. Nuclei are counterstained with YoYo-1 (green). Scale bar is 500 μm.

We first wanted to investigate how aging and exposure to EE might affect functional interactions between different cortical regions. Thus, we started our study by looking for monosynaptic corticocortical connections between the primary visual cortex (V1) and other sensory areas, by means of stereotaxic injections of the Cholera Toxin β subunit (CTB). Attention was paid to avoid spilling of the tracer to adjacent, higher-order visual areas; moreover, we verified that the injections remained confined to the core of V1 (Figure 1A).

Analysis of serial coronal brain sections revealed a monosynaptic connection between V1 and the primary auditory cortex (A1). In the hemisphere ipsilateral to the injected V1, labeled cells were located in layers IV–VI of A1, with a few scattered cells in layers II–III (Figure 1B, left panels). In the contralateral hemisphere, A1 neurons projecting to V1 were less abundant and preferentially located in layers V–VI (Figure 1B, right panels). No significant differences in the density and localization of CTB-positive neurons between EE and standard reared (SC) animals were found (data not shown).

### EE modulates the spectral power of local field potentials in V1 and A1

**Figure 2:**
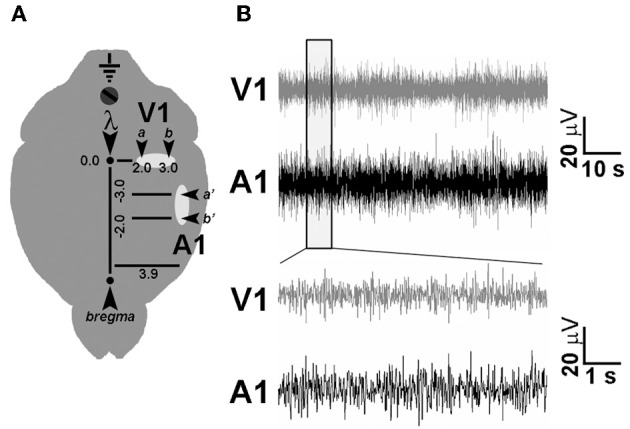
**Chronic electrode implant for LFP recordings. (A)** Schematic diagram showing electrode placement for LFP recording in V1 and A1. A couple of nichrome wire electrodes (arrowheads) were placed in each cortical area at the indicated stereotaxic coordinates to perform differential electrophysiological recordings. **(B)** Representative LFP traces recorded using chronic electrode implants in freely moving, awake mice.

After identifying a monosynaptic connection between A1 and V1, we asked whether EE could modulate (i) the profile of local activities in these two primary sensory areas and (ii) the interactions between them, by performing multichannel recordings of local field potentials (LFPs) from chronically implanted electrodes in enriched (EE) and standard-condition reared (SC) mice. The layout of electrode placement is shown in Figure 2. In order to assess possible age-dependent differences in the effect of EE, LFP recordings were performed not only in aged (EE-OLD and SC-OLD groups) but also in young mice (EE-YOUNG and SC-YOUNG groups).

We first evaluated the impact of EE on local electrical activity in V1 and A1 by calculating the Fourier transform of LFP traces. Fourier analysis was first made on the whole frequency range (0.3–45 Hz). We found no difference in the total spectral power (PWT) between EE-OLD, SC-OLD, EE-YOUNG and SC-YOUNG mice (data not shown), thus ruling out the possibility of variations among the four groups due to a global shifting of cortical activity out of the sampled frequency range.

**Figure 3:**
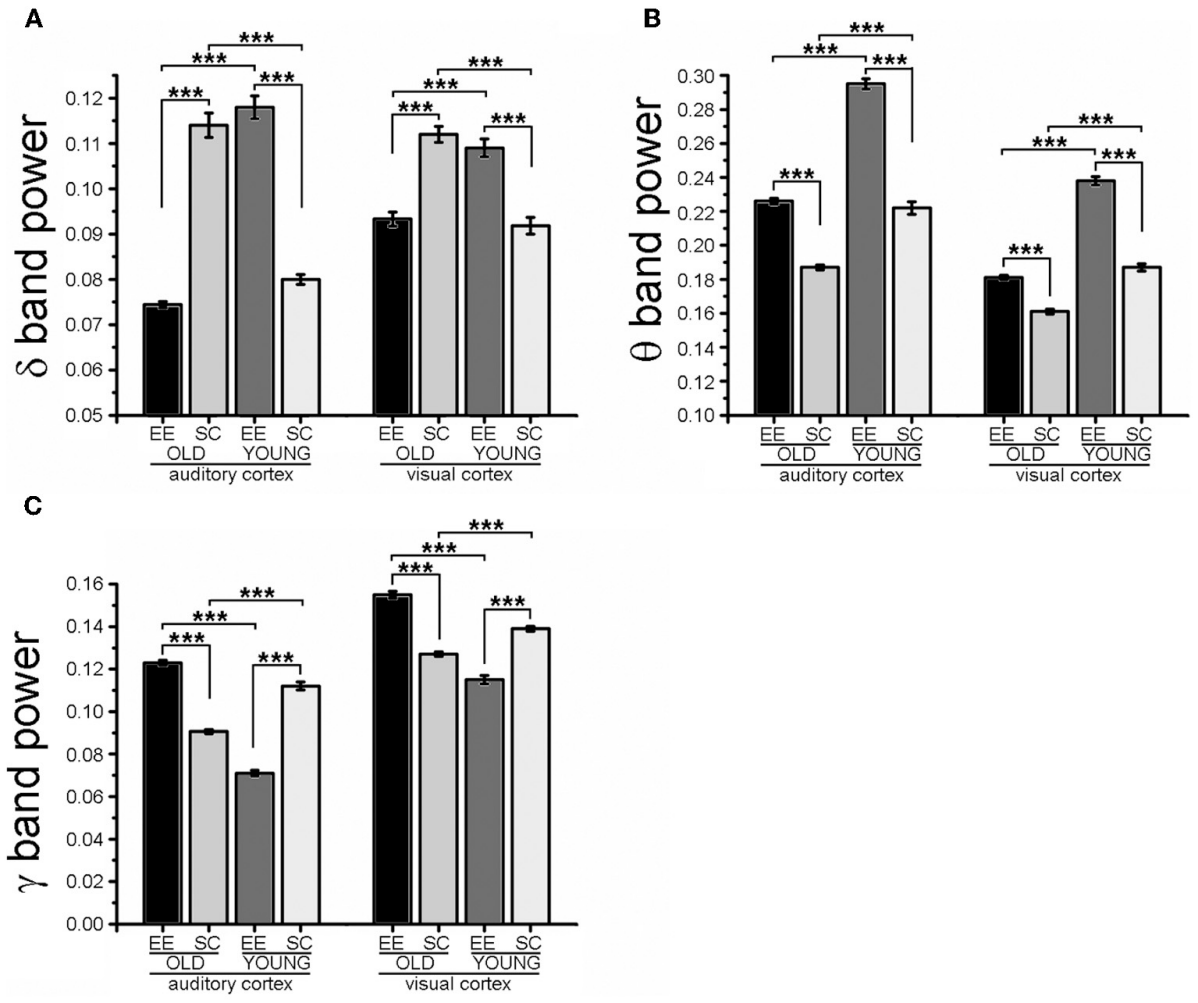
**Fourier analysis of spectral power in the δ, θ, and low-γ EEG bands**. The histograms show the results obtained with Fourier analysis in order to compute the spectral power of LFP in the δ **(A)**, θ **(B)** and low-γ **(C)** bands for auditory (A1) and visual (V1) cortices. For each cortical area, spectral powers for EE-OLD, SC-OLD, EE-YOUNG and SC-YOUNG groups were compared and statistical significance was assessed with Three-Way ANOVA followed by Holm-Sidak *post-hoc* test (^***^p < 0.001). **(A)** In the δ band, a statistically significant interaction between rearing condition, age and cortical area was found (Three-Way ANOVA, rearing × age × area interaction, *p* < 0.001). EE-OLD mice displayed lower LFP power than SC-OLD animals (rearing × age interaction, *p* < 0.001 and Holm-Sidak *post-hoc* test, *p* < 0.001 for both A1 and V1), whereas the opposite was observed when comparing the EE-YOUNG and SC-YOUNG groups (Holm-Sidak *post-hoc* test, *p* < 0.001 for both A1 and V1). **(B)** In the θ band, a statistically significant interaction between rearing condition and age was found (Three-Way ANOVA, rearing × age interaction, *p* < 0.001) and EE-OLD animals had higher LFP power than SC-OLD mice (Holm-Sidak *post-hoc* test, *p* < 0.001). This was similar to EE-YOUNG mice, which had increased θ band power in comparison to SC-YOUNG mice (Holm-Sidak *post-hoc* test, *p* < 0.001). **(C)** In the low-γ band, a statistically significant interaction between rearing, age and cortical area was found (Three-Way ANOVA, rearing × age × area interaction, *p* < 0.001) and EE-OLD animals had higher LFP power with respect to SC-OLD mice (rearing × age interaction, *p* = 0.004 for A1 and *p* < 0.001 for V1; Holm-Sidak *post-hoc* test, *p* < 0.001 for both A1 and V1). On the other hand, EE-YOUNG animals displayed decreased low-γ band power with respect to SC-YOUNG animals (Holm-Sidak *post-hoc* test, *p* < 0.001 for both A1 and V1).

Then, we analyzed in detail specific frequency bands and we found a clear effect of age: indeed, aged mice (SC-OLD) displayed a significantly higher spectral power in the δ band and a significantly lower power in the θ, α, and low-γ bands with respect to young mice (SC-YOUNG) in both A1 and V1 (Figure 3 and Figure S1B). In the β band, the spectral power was significantly decreased only in V1 (Figure S1B).

Exposure to EE affected the spectral power of all frequency bands. More in detail, the spectral power of the δ band was decreased in EE-OLD mice in comparison to SC-OLD mice. Of note, an opposite effect was observed when comparing the EE-YOUNG and SC-YOUNG groups (Figure 3A). θ and low-γ band spectral powers were increased with respect to SC-OLD mice in both A1 and V1 (Figures 3B,C); similar results were obtained when α and β bands were analyzed (Figures S1A,B). Interestingly, the increases in θ and α band power reproduce the effect of EE on YOUNG mice (Figure 3B and Figure S1A), whereas the spectral powers of β and low-γ bands are decreased when young mice are subjected to EE (Figure 3C and Figure S1B).

### EE affects cross-modal interactions between V1 and A1

**Figure 4:**
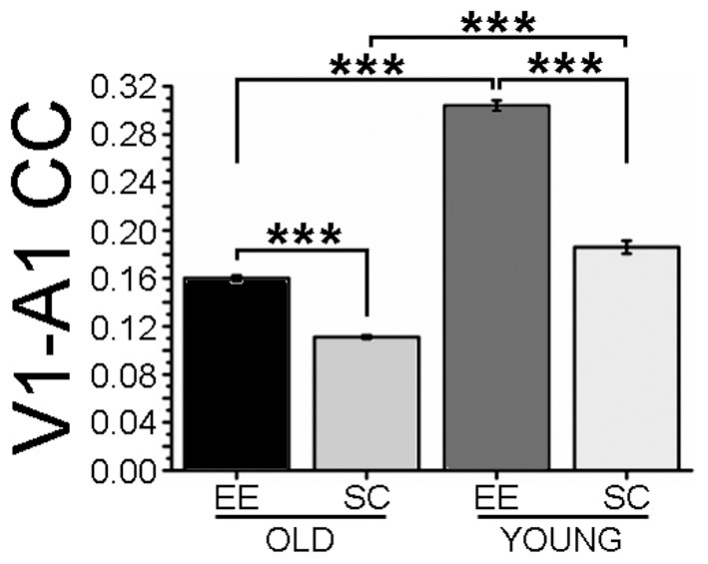
**Cross-correlation of LFPs recorded in visual and auditory cortices**. The histogram shows cross-correlation (CC) between LFP electrical activity recorded in visual and auditory cortices. CC values for the EE-OLD, SC-OLD, EE-YOUNG and SC-YOUNG groups were compared with Two-Way ANOVA (rearing × age interaction, *p* < 0.001) followed by Holm-Sidak *post-hoc* test (^***^p < 0.001). In both old and young mice, EE induced an increase in CC compared to SC controls.

We next investigated the impact of EE on crossmodal cortical interactions, by performing a cross correlation (CC) analysis between LFPs recorded in V1 and A1. We first computed CC of the whole frequency range of LFP signals (0.3–45 Hz). We found that normal aging resulted in a dramatic reduction of coupling between A1 and V1 (compare SC-OLD with SC-YOUNG, Figure 4); EE was effective to partly counteract this loss of functional connectivity in aged mice, resulting in a significantly higher CC in comparison to age-matched SC animals (Figure 4).

To look for the effects of EE on specific EEG frequency components, CC for the δ, θ, α, β, and low-γ bands were computed. CC was lower in SC-OLD than in SC-YOUNG mice for each band, with a less pronounced difference in the δ band (Figure S2). Significant increases in CC were observed in EE compared to SC mice across all frequency bands (Figures S2A–D), both for OLD and YOUNG groups. Notably, in young animals the effect of EE on the low-γ band was less pronounced, although still statistically significant (Figure S2E). It is worth noting that CC remained lower in EE-OLD than in EE-YOUNG mice in all bands, with the exception of the low-γ band, with EE-OLD mice showing a juvenile-like level of CC, albeit still significantly lower than in EE-YOUNG mice (Figure 4 and Figure S2).

### Modulation of the intracortical excitation/inhibition balance by EE

The effects of EE on the Fourier spectrum profiles and on visual-auditory cross-correlation prompted us to investigate whether these phenomena could be associated with the activation of molecular factors involved in neural plasticity processes. We focused our attention on the balance between cortical excitation and inhibition, a well-established factor for plasticity modulation in the adult brain.

**Figure 5:**
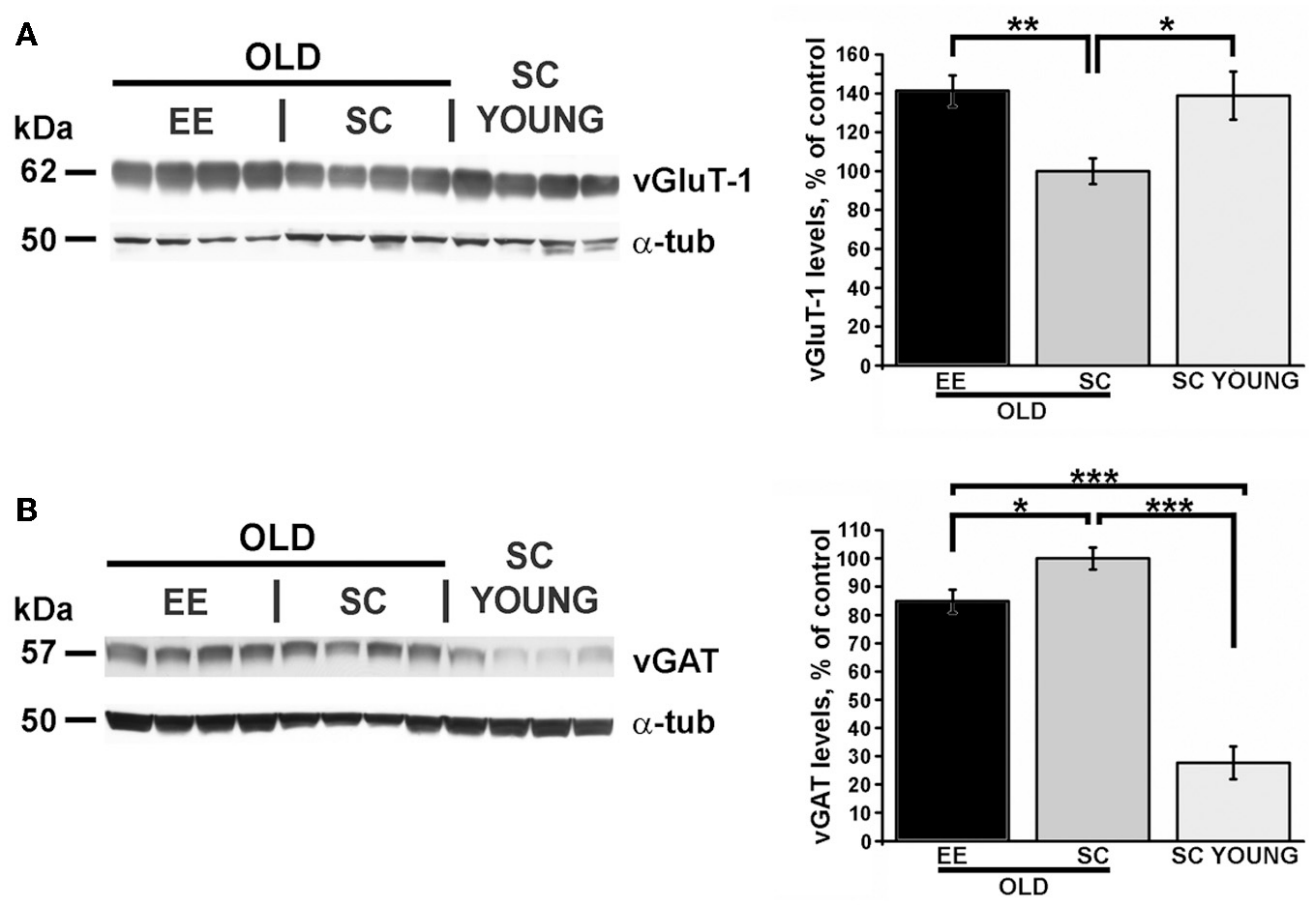
**EE affects excitation and inhibition in the cerebral cortex of aged and young mice. (A)** Left, representative immunoblotting and right, quantification of the GluT-1 glutamate transporter expression. While the SC-OLD group displayed a reduced expression level than SC-YOUNG mice, this reduction was completely counteracted in EE-OLD animals (One-Way ANOVA, *p* = 0.003, followed by Tukey's *post-hoc* test, ^*^p = 0.035, ^**^p = 0.004). **(B)** Left, representative immunoblotting and right, quantification of the vGAT GABA transporter expression. While the SC-OLD group displayed a increased expression level compared to SC-YOUNG mice, this increase was partially counteracted in EE-OLD animals (One-Way ANOVA, *p* < 0.001, followed by Tukey's *post-hoc* test, ^*^p = 0.041, ^***^p < 0.001).

We performed Western blots to quantify the expression of vGluT-1, the cerebral cortex-specific isoform of the vesicular glutamate transporter (Nakamura et al., 2005) and vGAT, the vesicular GABA transporter (Wang et al., 2009). Representative images of typical blots are shown in the left panels of Figure 5. Expression of vGluT-1 was significantly higher in the cerebral cortex of EE-OLD mice compared to SC-OLD controls (Figure 5A). No difference in cortical vGluT-1 levels was present between SC-YOUNG and EE-OLD mice. On the other hand, we found a significant reduction in cortical vGAT levels in EE-OLD mice compared to SC-OLD mice (Figure 5B). SC-YOUNG mice displayed the lowest vGAT level, whereas the highest value was observed in the SC-OLD group (Figure 5B). Thus, EE partly counteracted age-related alterations in glutamatergic and GABAergic markers, restoring a juvenile-like excitation/inhibition ratio in the cerebral cortex.

### Environmental enrichment modulates levels of soluble amyloid beta oligomers

Deposition of amyloid-beta protein (Aβ) in the brain parenchyma is not an exclusive prerogative of AD, but occurs also during normal aging (Bouras et al., 1994). To investigate whether EE affects Aβ levels in the cerebral cortex of aged mice, we quantified the expression levels of various soluble Aβ oligomer isoforms in both SC-OLD and EE-OLD mice.

**Figure 6:**
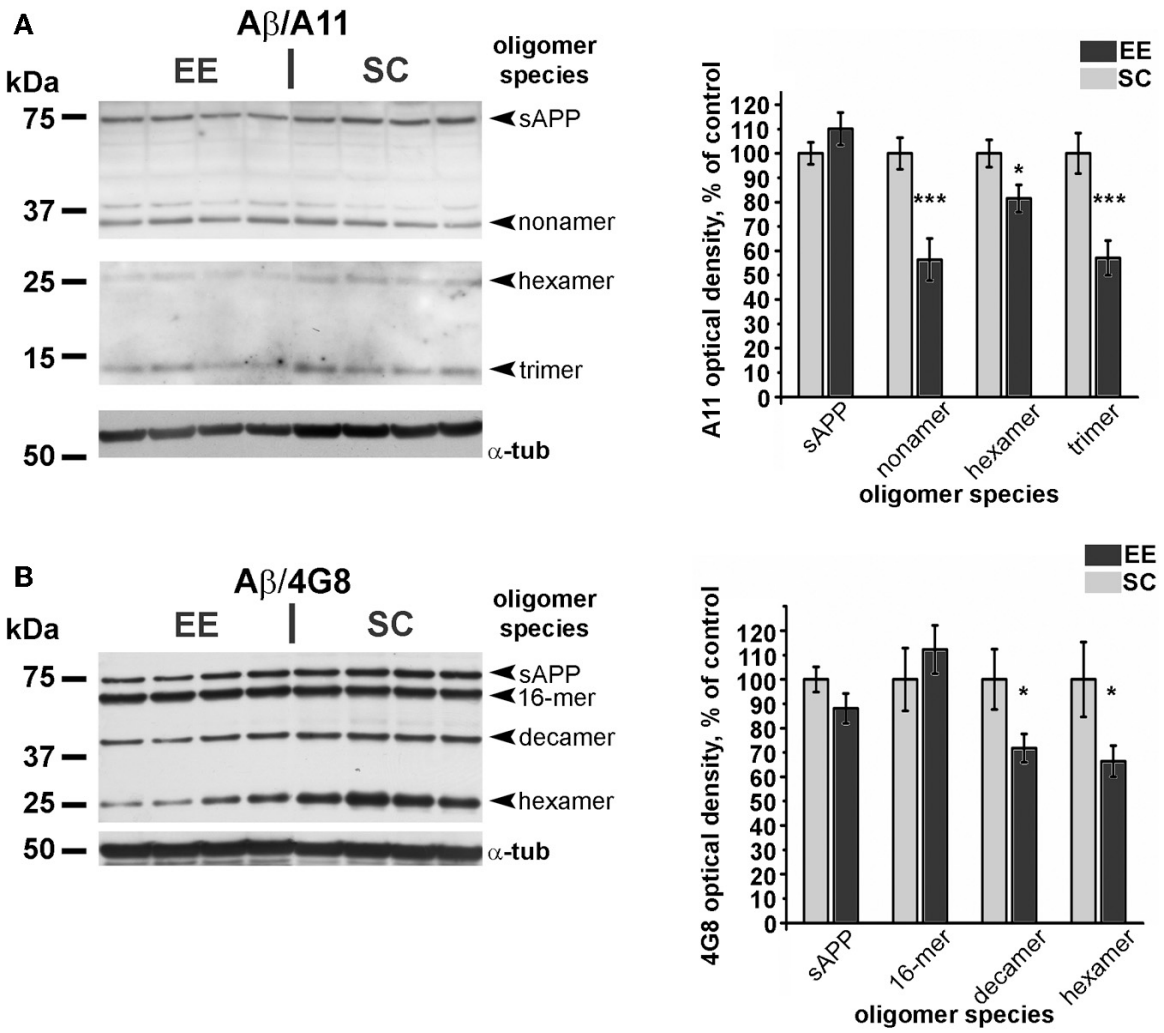
**Decrease of amyloid β oligomers in aged enriched mice. (A)** Left, representative immunoblotting and right, quantification of the expression of Aβ oligomers probed with the A11 polyclonal antibody. **(B)** Left, representative immunoblotting and right, quantification of expression of Aβ oligomers probed with the 4G8 monoclonal antibody. In both cases, optical density of autoradiographic bands mice was normalized as percentage of the value observed in SC-OLD mice. Statistical significance for each oligomer band was assessed using Student's *t*-test (^*^p < 0.05 and ^***^p < 0.001).

Since Aβ aggregates are observed across a wide range of molecular weights, with specific immunoreactivity profiles to antibodies commonly used in western blotting (Lesne et al., 2006), we performed Western blot analyses using two different antibodies, namely A11 and 4G8 (Table 1). These antibodies were selected because of their reactivity against mouse endogenous Aβ and basing on existing literature (Lesne et al., 2006). In our experimental conditions, we could detect Aβ oligomer isoforms ranging from trimers (12 kDa) to 16-mers (64 kDa), with only small variations in different primary antibody assays (Figure 6) and with consistent results deriving from quantification of the two independent blotting experiments. Our analysis revealed a significant decrease in the levels of low-molecular weight Aβ oligomers, specifically in the 12–36 kDa range, in EE-OLD mice compared to controls (Figure 6). The differentially expressed blot bands corresponded to trimers, hexamers, nonamers, and decamers (Figure 6). We did not observe any significant difference in the expression of high-molecular weight Aβ oligomers (approximately 64 kDa, 16-mers) in EE-OLD mice compared to SC-OLD animals.

**Figure 7:**
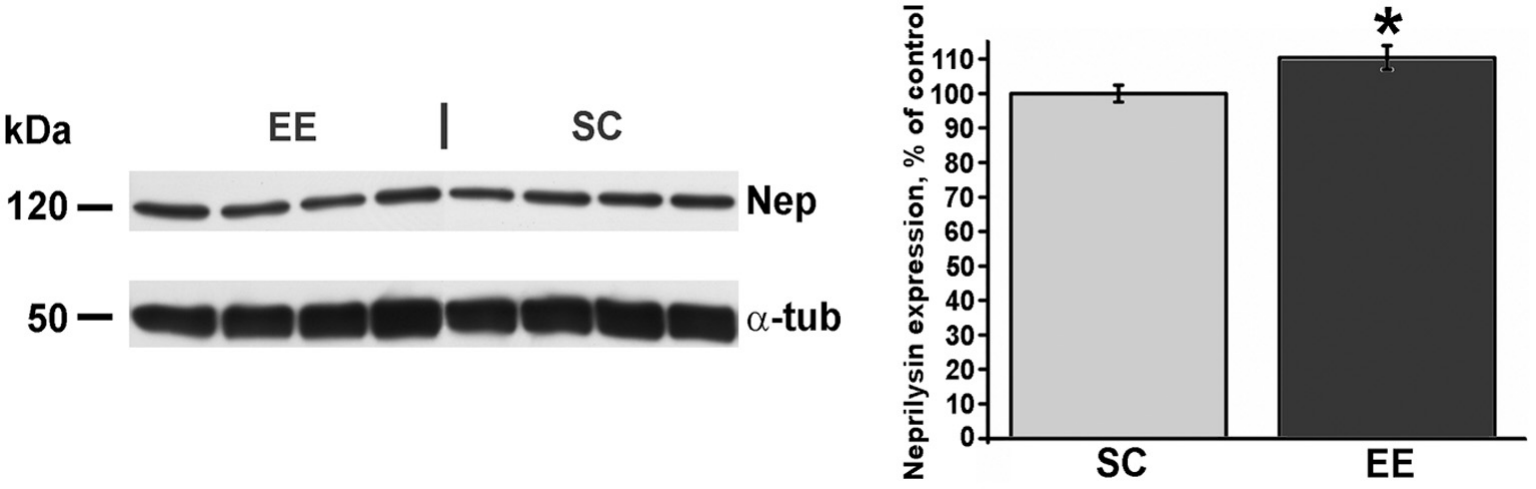
**Higher expression of neprilysin in aged enriched mice**. Left, representative immunoblotting and right, quantification of neprilysin abundance in the cerebral cortex of EE-OLD mice and SC-OLD mice. The values are normalized as percentage of the optical density of autoradiographic bands in SC-OLD mice. EE-OLD mice show a statistically significant increase in cortical neprilysin expression (Student's *t*-test, ^*^p < 0.05).

A reduced expression of cortical Aβ oligomers in EE mice could be derived from an enhancement in the processes involved in Aβ catabolism (Iwata et al., 2001). To test this possibility, we quantified via Western blot the cortical expression of neprilysin, one of the key enzymes for Aβ clearance in the brain parenchyma. Quantification of immunoblots revealed a statistically significant increase of neprilysin expression in EE-OLD mice compared to SC-OLD controls (Figure 7).

## Discussion

EE has been shown to ameliorate the pathological phenotype of several murine transgenic models of AD (Lazarov et al., 2005; Nithianantharajah and Hannan, 2006); however, its impact on physiological aging in laboratory rodents has received less attention. To fill this gap, we addressed the effects of a brief period of EE (one month) on aged (17 months) wild-type mice. In particular, we were interested to clarify how EE can impact brain physiology in the absence of overt pathological alterations. We analyzed the consequences of EE on local activity and functional coupling of primary sensory areas—i.e., primary auditory (A1) and visual cortices (V1)—and correlated the observed changes with the levels of cortical amyloid β protein (Aβ) soluble oligomers and neprilysin, as well as the expression of the neural plasticity markers vGluT-1 and vGAT.

Aging affects not only local activity of cortical areas, but also corticocortical interactions, in particular between sensory cortices (Golob et al., 2001). EE is a complex manipulation that has a global influence on the central nervous system, since it combines motor stimulation with social interaction and provides multimodal sensory inputs, spanning from the exploration of novel objects to periodical changes in the arrangement of the rearing space. Thus, EE is very likely to affect neural activity and plasticity in multiple brain areas and also corticocortical connections. We analyzed changes in the local activities of V1 and A1 by computing the spectral power of LFP across the main EEG bands. Our findings show that EE counteracts the age-dependent shift of EEG spectral power toward lower frequencies, which typically results in a higher power in the δ band, an effect described also in elderly humans (Breslau et al., 1989; Rossini et al., 2007). Interestingly, opposite effects on the δ band were observed in young mice in response to EE, i.e., a shift toward lower frequencies. Moreover, EE-OLD mice displayed a higher power in the θ band. Notably, higher activity in the θ band is observed during navigation tasks in humans (Caplan et al., 2003) and one of the features of EE is the exposure to a wider and more complex rearing environment, which might enhance spatial exploration. An age-specific difference was also observed when the spectral power in the low-γ band was computed, with EE-OLD animals showing a higher power in comparison to SC-OLD mice, whereas EE-YOUNG mice displayed a lower spectral power in the same frequency band with respect to SC-YOUNG mice. Age-specific differences in the molecular pathways mediating the beneficial effects of EE on the pathophysiology of AD have been described in a mouse transgenic model (Herring et al., 2011). Moreover, key mediators of the acceleration in brain development observed when EE starts before birth (corresponding to our EE-YOUNG condition), have been shown to be (i) a higher concentration of IGF-1 both in the milk and in the newborn brain (Sale et al., 2007a; Guzzetta et al., 2009) and (ii) a precocious rise in the cortical levels of BDNF (Sale et al., 2004), which rely on enhanced maternal care (Cancedda et al., 2004). On the other hand, induction of plasticity by EE during adulthood has been shown to depend on a higher production of BDNF (Sale et al., 2007b) and IGF-1 as well (Maya-Vetencourt et al., 2012), but in this case physical exercise and exploration of novel objects appear to be responsible for the effect at the molecular level. Thus, different environmental stimuli appear to converge on the same molecular effectors both during immediate postnatal life and in adulthood.

It is tempting to speculate that the age-specific peculiarities emerging from Fourier analysis reflect differences in the mechanisms of EE action on neural activity of young and aged brain, possibly representing the functional signature of compensatory plasticity processes based on the recruitment of different neural circuits, as proposed by the “scaffolding hypothesis” of the aging brain (Park and Reuter-Lorenz, 2009). Interestingly, impaired brain connectivity has also been described to be related to the development of AD (D'Amelio and Rossini, 2012); thus, EE can be effective in counteracting the changes in neuronal activity that are associated to the development of age-related dementia.

Connections between V1 and A1 have been described in the cat, in the primate and in the prairie vole (Innocenti and Clarke, 1984; Falchier et al., 2002; Campi et al., 2009). Here, we showed a direct connection between V1 and A1 for the first time in the mouse. Previous electrophysiological findings demonstrated the existence of auditory responses in visual neurons (Morrell, 1972; Fishman and Michael, 1973; Iurilli et al., 2012), but the global interaction between A1 and V1 had not been studied before. We found that aging is associated to a decrease in cross-correlation between A1 and V1 activity. The V1-A1 corticocortical interaction was responsive to EE, with aged EE animals displaying higher V1-A1 CC, thus reproducing the situation observed in young EE mice. Since the CC ratio between EE-OLD and SC-OLD mice was very similar to that between EE-YOUNG and SC-YOUNG animals (respectively, 1.44 and 1.63), the impact of EE appears to persist unaltered with aging.

Some differences between aged and young mice emerged when CC was analyzed more in detail by selecting specific EEG frequency bands. Since high-frequency EEG activity is associated with complex brain functions, such as learning and cognition (Herrmann et al., 2010), the effect of EE on higher-frequency bands, in particular the β and low-γ bands, in old mice could represent a physiological correlate of a reduction in the cognitive decline associated to aging. On the other hand, the marginal change in the low-γ band of young animals could reflect a saturation of activity in this frequency range.

It can be hypothesized that the increase in beta and gamma power reflects a status of heightened synaptic excitability, as has also been postulated for humans (Ferreri et al., 2013). This is consistent with the variations in the expression of synaptic plasticity markers, in particular with the increased abundance of vGluT-1 (see below).

It is worth noting that LFP signals recorded from young animals showed less variability in comparison to those obtained from aged animals. Indeed, standard errors of mean (s.e.m.) were comparable between the two ages, although the *n* of young groups was lower than aged groups (see Materials and Methods). We may hypothesize that this reflects a combination of interindividual differences in (i) the outcome of the physiological aging process and (ii) the response to EE in late adulthood.

Another potential factor influencing the interaction between A1 and V1 could be a possible effect of EE on age-related presbyopia and presbyacusis, since a progressive stiffening of the lens (Baradia et al., 2010) and age-related hearing loss (Johnson et al., 1997) have been described in C57BL/6J mice. Further experiments will be required to address this point.

The effect of EE on local LFP activity in V1 and A1 and on corticocortical interactions between these two primary sensory cortices pointed to neural plasticity phenomena (Bavelier and Neville, 2002). We looked for a molecular correlate of functional changes by analyzing the expression of vGluT-1 and vGAT, which are reliable markers to probe the global excitatory and inhibitory tones of the brain (Mainardi et al., 2010a,b). It is worth noting that changes in intracortical inhibition have been associated also with physiological brain aging. However, studies in humans and animal models have shown changes in either direction (Luebke et al., 2004; McGinley et al., 2010; Schmidt et al., 2010; Marneweck et al., 2011; Cheng and Lin, 2013). On the other hand, the role of excitation has not been studied in detail. We provide evidence that EE is able to increase the cortical excitatory tone, in addition to decreasing inhibition. Notably, aged SC mice displayed the higher degree of cortical inhibition, whereas the lowest degree was observed in young SC mice, with intermediate levels in aged EE mice. The effect of EE on cortical excitation was stronger, and vGluT-1 expression reached juvenile-like values.

A key histopathological sign of AD is the accumulation in the brain parenchyma of Aβ oligomers, which are found in a wide range of molecular weights, ranging from 4 kDa (monomers) to about 65 kDa (Lesne et al., 2006). However, Aβ deposition takes place also during healthy aging (Bouras et al., 1994). There is an increasing amount of literature supporting a role of low-molecular weight Aβ oligomers in the generation of synaptic dysfunction and plasticity deficits associated with dementia (Pham et al., 2010; Sokolow et al., 2012). Here, we used Western blotting to quantify the expression of various endogenous Aβ isoforms and found a significant decrease in low-molecular weight oligomers (trimers to decamers) in aged mice subjected to EE.

In our experimental conditions, we were unable to detect Aβ monomers and dimers. A possible explanation is that we worked in physiological conditions, whereas the majority of literature relies on transgenic models that overexpress mutated variants of AD-related genes (Ashe and Zahs, 2010) and accumulate high amounts of Aβ. Indeed, the equilibrium of different Aβ oligomers in aged but otherwise healthy animals can be different from pathological models. It is also worth noting that, due to three mutations in the aminoacilyc sequence, murine Aβ does not aggregate to form amyloid plaques (Jankowsky et al., 2007). Nonetheless, soluble endogenous Aβ can be detected in brain preparations from wild-type mice using appropriate assays (Teich et al., 2013). Thus, despite different aggregation dynamics, endogenous Aβ can contribute to age-related physiological and biochemical modifications occurring in the brain of wild-type mice.

We also analyzed the expression of neprilysin, an enzyme with protease activity that is the main responsible for removal of Aβ from cerebral tissue (Iwata et al., 2001). Since overexpression of neprilysin in the J20 pathological model decreases the abundance of Aβ (Meilandt et al., 2009), our finding that EE mice display a higher expression of neprilysin suggests that the reduction in cortical Aβ oligomers can be due, at least in part, to improved clearance from the cortical tissue. Consistently, an analogous correlation between lower Aβ burden and increased neprilysin activity has been observed also when transgenic mouse harboring the APP Swedish and the Presenilin-1 Δ E9 mutations were subjected to EE (Lazarov et al., 2005).

Taken together, our findings indicate that a brief exposure to enriched conditions in aged mice can induce profound changes in cortical functionality, decreasing levels of Aβ oligomers and promoting neural plasticity. These results provide a functional and biochemical basis for understanding how enriched environmental stimuli can improve brain health and functioning during aging.

## Author contributions

Conceived and designed experiments, Marco Mainardi, Nicoletta Berardi, Matteo Caleo, Alessandro Sale, Lamberto Maffei; performed experiments, Marco Mainardi, Alessandro Sale; analyzed the data, Marco Mainardi, Angelo Di Garbo; wrote the manuscript, Marco Mainardi, Angelo Di Garbo; revised the manuscript, Nicoletta Berardi, Alessandro Sale, Matteo Caleo, Lamberto Maffei.

### Conflict of interest statement

The authors declare that the research was conducted in the absence of any commercial or financial relationships that could be construed as a potential conflict of interest.

## Supplementary material

Figure S1 **Fourier analysis of spectral power in α and β EEG bands**. The histograms show results of the Fourier analysis used to compute the spectral power of LFP signals in the α **(A)** and β **(B)** bands for visual and auditory cortices. For each cortical area, spectral powers for EE-OLD, SC-OLD, EE-YOUNG, and SC-YOUNG groups were compared. Statistical significance was assessed with Three-Way ANOVA followed by Holm-Sidak *post-hoc* test (^*^*p* = 0.032; ^***^*p* < 0.001). **(A)** In the α band, a statistically significant interaction between rearing, age and cortical area was found (Three-Way ANOVA, rearing × age × area interaction, *p* = 0.004) and EE-OLD mice had higher LFP power in comparison to SC-OLD mice (rearing × age interaction, *p* < 0.001 for A1 and *p* = 0.002 for V1; Holm-Sidak *post-hoc* test, *p* < 0.001 for both A1 and V1), which was similar to what observed when comparing the EE-YOUNG and SC-YOUNG groups (Holm-Sidak *post-hoc* test, *p* < 0.001 for both A1 and V1). **(B)** In the β band, a statistically significant interaction between rearing, age and cortical area was found (Three-Way ANOVA, rearing × age × area interaction, *p* = 0.001) and EE-OLD animals showed higher LFP power compared to SC-OLD animals (rearing × age interaction, *p* < 0.001 and Holm-Sidak *post-hoc* test, *p* < 0.001 for both A1 and V1); on the other hand, EE-YOUNG mice had decreased LFP power with respect to SC-YOUNG mice (Holm-Sidak *post-hoc* test, *p* < 0.001 for both A1 and V1).

Figure S2 **Cross-correlation of LFPs recorded in visual and auditory cortices in the main EEG bands**. The histograms show cross-correlation (CC) between LFP electrical activity recorded in visual and auditory cortices for the δ **(A)**, θ **(B)**, α **(C)**, β **(D)** and low-γ **(E)** bands. CC for the EE-OLD, SC-OLD, EE-YOUNG, and SC-YOUNG groups were compared with Two-Way ANOVA (rearing × age interaction, **(A)** *p* < 0.001, **(B)** *p* < 0.001, **(C)** *p* < 0.001, **(D)** *p* < 0.001, **(E)** *p* < 0.001) followed by Holm-Sidak *post-hoc* test (^*^*p* < 0.05, ^**^*p* = 0.007 and ^***^*p* < 0.001). In both old and young mice, EE induced an increase in CC compared to SC controls.
