# Supplementary figures and images for: Environmental enrichment strengthens corticocortical interactions and reduces amyloid-β oligomers in aged mice
Source: Front Aging Neurosci. 2014 Jan 23;6:1. doi: 10.3389/fnagi.2014.00001 (PMC3899529; doi:10.3389/fnagi.2014.00001)

**A**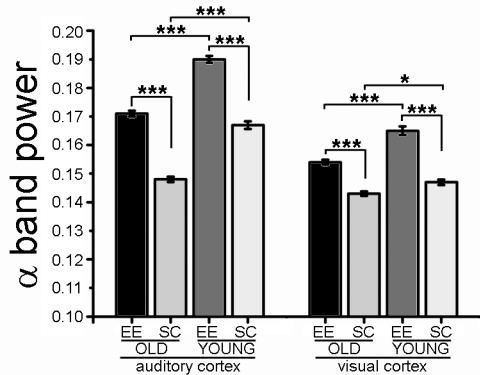**B**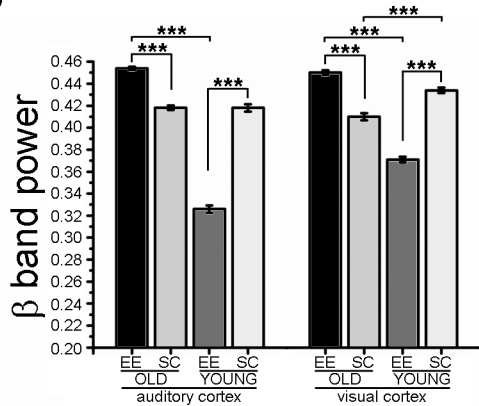

Supplement: Figure S1 — Fourier analysis of spectral power in α and β EEG bands. The histograms show results of the Fourier analysis used to compute the spectral power of LFP signals in the α (A) and β (B) bands for visual and auditory cortices. For each cortical area, spectral powers for EE-OLD, SC-OLD, EE-YOUNG, and SC-YOUNG groups were compared. Statistical significance was assessed with Three-Way ANOVA followed by Holm-Sidak post-hoc test (*p = 0.032; ***p < 0.001). (A) In the α band, a statistically significant interaction between rearing, age and cortical area was found (Three-Way ANOVA, rearing × age × area interaction, p = 0.004) and EE-OLD mice had higher LFP power in comparison to SC-OLD mice (rearing × age interaction, p < 0.001 for A1 and p = 0.002 for V1; Holm-Sidak post-hoc test, p < 0.001 for both A1 and V1), which was similar to what observed when comparing the EE-YOUNG and SC-YOUNG groups (Holm-Sidak post-hoc test, p < 0.001 for both A1 and V1). (B) In the β band, a statistically significant interaction between rearing, age and cortical area was found (Three-Way ANOVA, rearing × age × area interaction, p = 0.001) and EE-OLD animals showed higher LFP power compared to SC-OLD animals (rearing × age interaction, p < 0.001 and Holm-Sidak post-hoc test, p < 0.001 for both A1 and V1); on the other hand, EE-YOUNG mice had decreased LFP power with respect to SC-YOUNG mice (Holm-Sidak post-hoc test, p < 0.001 for both A1 and V1). [file Presentation1.PDF]

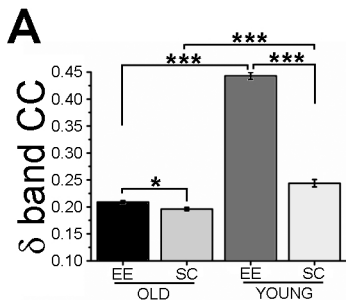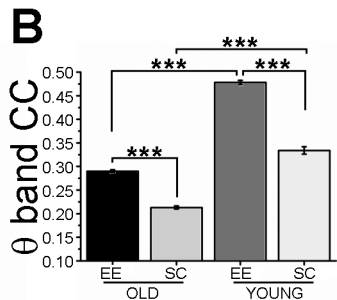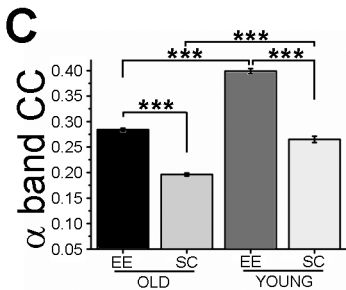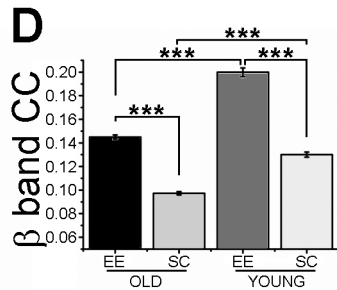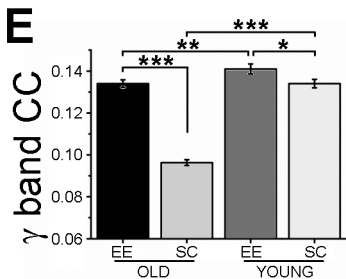

Supplement: Figure S2 — Cross-correlation of LFPs recorded in visual and auditory cortices in the main EEG bands. The histograms show cross-correlation (CC) between LFP electrical activity recorded in visual and auditory cortices for the δ (A), θ (B), α (C), β (D) and low-γ (E) bands. CC for the EE-OLD, SC-OLD, EE-YOUNG, and SC-YOUNG groups were compared with Two-Way ANOVA (rearing × age interaction, (A) p < 0.001, (B) p < 0.001, (C) p < 0.001, (D) p < 0.001, (E) p < 0.001) followed by Holm-Sidak post-hoc test (*p < 0.05, **p = 0.007 and ***p < 0.001). In both old and young mice, EE induced an increase in CC compared to SC controls. [file Presentation2.PDF]
